# Measured voluntary avoidance behaviour during the 2009 A/H1N1 epidemic

**DOI:** 10.1098/rspb.2015.0814

**Published:** 2015-11-07

**Authors:** Jude Bayham, Nicolai V. Kuminoff, Quentin Gunn, Eli P. Fenichel

**Affiliations:** 1 Yale School of Forestry and Environmental Studies, Yale University, 195 Prospect Street, New Haven, CT 06511, USA; 2 College of Agriculture, California State University, Chico, Chico, CA 95926, USA; 3 Department of Economics, Arizona State University, Tempe, AZ 85287, USA

**Keywords:** avoidance behaviour, A/H1N1, social distancing

## Abstract

Managing infectious disease is among the foremost challenges for public health policy. Interpersonal contacts play a critical role in infectious disease transmission, and recent advances in epidemiological theory suggest a central role for adaptive human behaviour with respect to changing contact patterns. However, theoretical studies cannot answer the following question: are individual responses to disease of sufficient magnitude to shape epidemiological dynamics and infectious disease risk? We provide empirical evidence that Americans voluntarily reduced their time spent in public places during the 2009 A/H1N1 swine flu, and that these behavioural shifts were of a magnitude capable of reducing the total number of cases. We simulate 10 years of epidemics (2003–2012) based on mixing patterns derived from individual time-use data to show that the mixing patterns in 2009 yield the lowest number of total infections relative to if the epidemic had occurred in any of the other nine years. The World Health Organization and other public health bodies have emphasized an important role for ‘distancing’ or non-pharmaceutical interventions. Our empirical results suggest that neglect for voluntary avoidance behaviour in epidemic models may overestimate the public health benefits of public social distancing policies.

## 1. Introduction

Managing infectious disease is among the foremost challenges for public health policy. The World Health Organization and other public health bodies have emphasized an important role for ‘social distancing’ or non-pharmaceutical interventions such as school and workplace closure [1]. Indeed, studies support that distancing policy can effectively mitigate disease spread by reducing contact between susceptible and infected individuals [2–4]. However, distancing policy can impose large economic and social costs [5–8]. In order to understand the public health benefits of social distancing policies, we must establish a behavioural baseline during an epidemic—the public health outcomes resulting from private actions of individuals to reduce their risk of infection [9].

Economic epidemiology theory suggests that susceptible individuals may forgo beneficial contacts in order to reduce the probability of contracting a costly infectious disease [7,10–13]. Following standard epidemiological theory, voluntary avoidance behaviour mitigates disease transmission and implies a dynamic feedback between humans and pathogens over the course of an epidemic [14]. We study whether individuals engaged in epidemiological avoidance behaviour during the 2009 A/H1N1 epidemic, and if the magnitude of individual behavioural shifts was of sufficient magnitude to alter epidemiological dynamics in the USA. This enables us to quantify the approximate size of adaptive behavioural feedbacks on public health outcomes.

Several studies have used simulation to illustrate the potential public health benefits of avoidance behaviour [15,16]. Empirical efforts to quantify individuals' responses to infectious disease risk are often based on one-off surveys in the wake of the epidemic, but not coupled with epidemiological dynamics [17–20], or infer potential avoidance behaviour *ex post* from observed epidemic outcomes [21–23]. While these studies provide empirical insights into the role of avoidance behaviour during an epidemic, no study has quantified avoidance behaviour based on observable time-use data and coupled that behavioural shift with an epidemiological model to provide an empirical estimate of the public health consequences of avoidance behaviour. Our study bridges methods by using surveyed behavioural data and reported epidemic data in an epidemiological model to fill this important gap in the literature, thereby complementing insights from models targeted at other aspects of the epidemic [24–29]. We estimate voluntary avoidance behaviour during the 2009 A/H1N1 (swine) flu epidemic using a detailed dataset with daily observations on how Americans spent their time between 2003 and 2012. We use this estimate to quantify the public health impacts of such avoidance behaviour, and we show that individual voluntary avoidance behaviour was of sufficient magnitude to meaningfully alter disease dynamics and impact transmission of the A/H1N1 influenza virus.

## 2. Methods

### (a) Data

Data for this study come from multiple sources. Time-use data for the general population were compiled from the American Time-Use Survey (ATUS) (2003–2012) [24]. The ATUS is subsampled from the US Current Population Survey, which contains detailed demographic and socioeconomic information about respondents older than 15 years old and their family members (including children under 15 years of age). Survey respondents report a 24 h diary of activities, locations and accompanying persons for every minute of the day. We supplement the ATUS data with time-use data on children at school from the National Health and Activity Patterns Survey (NHAPS), a similar time-use survey that includes children under 15 years old [25]. The combined dataset consists of 146 331 respondents with sample weights that report an average of 16.1 activities per day (more details about this dataset are in the electronic supplementary material).

The weekly number of laboratory-confirmed cases at the national level were collected from Brammer *et al*. [26], who obtain data from the Centers for Disease Control and Prevention (CDC) Influenza Surveillance System. We used this measure of disease prevalence to capture the objective risk of spending time in public. US laboratory-confirmed cases peaked at 9734 during the week of 18–24 October 2009 (electronic supplementary material, figure S1). Data from Google Trends were used to measure the subjective risk of infection over the course of the epidemic. A detailed description of the Google search index is provided in the electronic supplementary material. Extreme weather data were collected from the National Oceanic and Atmospheric Administration National Climatic Data Center (NCDC) Storm Events Database and is described in detail in the electronic supplementary material [27]. Extreme weather includes daily information about storms and other significant weather phenomena, unusual weather activity that generates media attention, and significant meteorological events (record minimum and maximum temperature) at the county level. Extreme weather was used to control for additional time spent at home to avoid weather rather than flu risk.

### (b) Regression

We specify a series of fixed-effects regression models to test the hypothesis that individuals engaged in avoidance behaviour in response to subjective (media attention) and objective (laboratory-confirmed cases) measures of risk. The fixed-effects regression model is
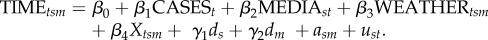


The variable TIME denotes the number of minutes spent at home (ATUS) and subscript *t* indexes date, and the subscripts *s* and *m* index state and month. Time spent at home is considered safer than in public microenvironments during an epidemic and is indeed the motivation for social distancing policy such as school closure. Moreover, 26% of US households consist of a single individual, which eliminates all household infection risk for this 26% of households [24]. We develop this argument further in the epidemic model section.

The variable MEDIA represents the Google search index, CASES represents the number of CDC laboratory-confirmed weekly cases, WEATHER represents instances of extreme weather, *X* is a vector of demographic characteristics described in the electronic supplementary material, and *d* and *a* are month and state dummy variables that form the fixed-effects model.

We estimate three models using state fixed effects (model 1), month fixed effects (model 2) and state-by-month fixed effects (model 3) to illustrate that our estimates are robust across model specifications (two additional models are presented in the electronic supplementary material to further examine model specification uncertainty). All regressions are based on 27 091 observations of ATUS from 2008 to 2010. The years 2008 and 2010 are pre- and post-epidemic periods that are believed to be most similar to the epidemic period and therefore serve as robust ‘control’ periods. Angrist & Pischke [28] suggest restricting such analysis to the most similar ‘control’ periods when samples are sufficiently large to do so. Summary statistics are reported in electronic supplementary material, table S2. All regression analyses were conducted in Stata v. 12.

### (c) Epidemic simulations

We construct an SIR compartmental model to develop a first approximations to counterfactual epidemics of the 2009 A/H1N1 outbreak [29–31]. We specify the set of differential equations governing the transmission dynamics as2.1
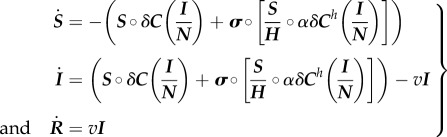
where ‘°’ and ‘–’ denote element by element multiplication and division. ***S***, ***I***, and ***R*** are *K* × 1 vectors of susceptible, infectious and recovered health classes where *K* is the number of subpopulations (e.g. age groups). ***N*** is a *K* × 1 vector of subpopulations in each segment. ***H*** is a *K* × 1 vector of the number of households in each subpopulation. ***C*** and ***C****^h^* are *K* × *K* public and household probabilistic contact matrices that describe the interaction between and individual in subpopulation *j* (rows) and subpopulation *k* (columns). ****σ**** is a *K* × 1 vector indicating the number of infected households where each element must be strictly between **0** and ***I***. *δ* is the disease-specific infectivity parameter, or conditional probability of transmission per minute of contact between a susceptible and infected individual. *α* is a scalar that adjusts the relative infectiveness of a contact minute in the home relative to one in public. 1/*v* is the average infectious period constant across classes.

Epidemic dynamics depend on time spent in public and household environments [32,33]. The first term in equation (2.1) captures public transmission and is the contact time analogous to common specifications. An individual makes potentially infectious contacts with household members if and only if there is at least one infected person in the household. The second terms capture within-household transmission in the infectious home environment. This model makes a number of conservative assumptions that inflate the within-household transmission. These assumptions will work to mask the epidemiological effects of individuals attempting to avoid infection by allocating more time to the household. We find significant effects of avoidance behaviour in spite of these assumptions. At any point in time, the expected number of susceptible individuals within the average household for each subpopulation is at most ***S***/***H***. This is an expectation across the entire population, but knowing that infected households must have at least one infectious individual and that household sizes are fixed implies the expected number of susceptible individuals in an infectious household must be less than ***S***/***H***, unless all households are infected. We approximate the number of infectious households in subpopulation *j* as *I*, which maximizes the potential for within-household transmission. These assumptions are conservative and overestimate within-household transmission because they allow the greatest number of households to be infected, imply a larger number of susceptibles in the infectious household environment than are truly at risk at home, and implicitly allow members of infectious households to ‘mix’ freely among infectious households, regardless of true home.

We assume *δ* is common to all population types. This assumption could be generalized with estimates of *δ* that are location and attribute class specific that are independent of behaviour. To the best of our knowledge, such estimates do not exist, because the multiplicative relationship between contact time and infectivity makes identification of location-specific *δ* difficult without imposing additional assumptions. Using age- or location-specific estimates of *δ* that did not control for contact time would confound our results.

While many detailed models of the H1N1 epidemic have been proposed [34–36], our work focuses on the behavioural responses and feedback to epidemiological dynamics. We use a relatively simple model in order to focus attention on the potential role of voluntary avoidance behaviour as a feedback mechanism. We model an epidemic over a short period of time such that births and deaths are negligible, a common assumption for influenza [37–39]. The model assumes that the entire population is susceptible prior to the introduction of the pathogen.

We simulate the epidemiological relevance of the avoidance behaviour estimated in the regression model using a homogeneous mixing model (*K* = 1). In the baseline case, public contact time is equal to the average of time spent in public in the ATUS (*C*_0_ = 316 min or 5.26 h per day) and remains constant throughout the epidemic (no avoidance). Alternatively, individuals respond to disease risk by shifting time in public to their household. Formally, 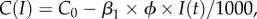 where *β*_1_ is the minutes of avoidance behaviour per 1000 cases estimated from the regression model and *ϕ* = 8.33% is the proportion of infected individuals confirmed through laboratory testing described in the electronic supplementary material. Likewise, 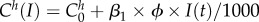 is the contact time the average individual experiences at home. We assume that the population has no memory and only responds to disease risk at time *t*, which yields conservative estimates of the epidemiological impact of avoidance behaviour. This infection-dependent contact rate is similar to the effective rate of transmission characterized by Funk *et al.* [15] to model avoidance behaviour as a function of information about disease risk.

While the relationship between time spent interacting in public and transmission may be complex and depend on many factors, *δ* can be interpreted as a first-order approximation of infectivity conditional on contact. We calibrate the conditional infectivity, *δ*, such that the maximum of the simulated prevalence path under avoidance behaviour equals the peak prevalence observed during the 2009 A/H1N1 outbreak, as estimated for 9734 national cases, which yields *δ* = 1.4 × 10^−3^. The simulation without avoidance then represents the possible epidemic outcomes had no individual engaged in avoidance behaviour. We set the household contact scalar to unity (*α* = 1). This assumption is based on a systematic review of the empirical literature on household transmission that finds no consistent patterns [40]. Cauchemez *et al*. [41] and House & Keeling [33] find that larger households do not appear to have greater within-home transmission, suggesting that a minute in proximity with an infected person within in a household is probably not qualitatively different from a minute spent with an infected person outside the household. We investigate the sensitivity of the results to this assumption in the electronic supplementary material.

We abstract from the patchy structure of the US population [42] and scale our mode to simulate flu dynamics for a single highly connected city. We assume a population of 4.1 × 10^6^ with 1.5 × 10^6^ households, representing a US city the size of the Phoenix–Mesa–Glendale Metropolitan Statistical Area (MSA). We choose Phoenix–Mesa–Glendale MSA, because there was substantial concern about the epidemic given its close geographical and cultural connection to Mexico—the epicentre of the H1N1 epidemic. We initialize the epidemic by introducing 33 infected individuals into the susceptible population (one in each subpopulation). Increasing the number of initially infected individuals accelerates the time until large-scale outbreak but has no effect on the avoidance results.

### (d) Probabilistic contact matrix

We provide an alternative test for the impact of avoidance behaviour on epidemic dynamics by constructing counterfactual epidemics via simulation based on probabilistic contact matrices (PCMs) derived from the ATUS for each year between 2003 and 2012 [43]. The PCMs specify the amount of time (excluding time asleep) an individual in one group is exposed to populations in other groups. We divide the population into 35 groups (bins) based on age and household size ({0–4, 5–12, 13–17, 18–24, 25–49, 50–64, 65+} × {1, 2, 3, 4, 5+}∈*P*), where set *P* is of length *K* (electronic supplementary material, figure S3). The age bins do not contain equal shares of the population. Rather, the bins are designed to balance the desire for a small number of groupings with our desire that the bins also reflect life stages most likely to influence behaviours such as school and employment time obligations (see the electronic supplementary material).

These PCMs capture the fact that individuals can modify their schedule to avoid potentially infected individuals as an alternative form of avoidance behaviour. For example, young adults may go to health clubs and gyms in the evening to socialize, whereas other adults may go early in the morning to avoid congestion. Our PCM construction approach captures an individual's reallocation of time throughout the day across many activities including time at home. We break each individual year from 2003 to 2012 into two periods and construct a total of 20 PCMs. The first period is 20 April–20 December, the period of the actual A/H1N1 outbreak during 2009. The second period is 1 January–19 April, the period prior to the 2009 A/H1N1 outbreak. For brevity, we refer to 20 April through 20 December as the outbreak period and 1 January through 19 April as the pre-outbreak period. Counterfactual simulations are conducted for each empirical PCM calculated from the ATUS and NHAPS data according to the SIR model. We provide more detail on the PCM methods in the electronic supplementary material.

### (e) Quantifying uncertainty

We employ Monte Carlo techniques to calculate confidence intervals around simulation results because of the nonlinearity in the SIR model and lack of closed-form solution. The fixed-effects regression yields a parameter estimate of *β*_1_ with distribution 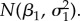 The regression parameter estimates form a Bayesian prior distribution from which we sample for each simulation of the SIR model. We simulate the SIR model 1000 times. In each simulation, the avoidance parameter is drawn from the distribution 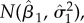 where the parameters are estimated in the regression. We report the 2.5 and 97.5 percentiles of the 1000 simulated results.

The construction of the PCM and subsequent epidemic simulations are deterministic. However, the ATUS is a stratified random sample of the US population, and thus sampling error exists. We employ a bootstrap approach to estimate standard errors for each element of the contact matrices as well as epidemic simulation outcomes (e.g. cumulative cases). We construct 1000 independent resamples with replacement of the ATUS dataset, each the size of the original dataset [44]. We sample at the respondent level and not the activity level, so if an individual was selected, his or her entire 24 h diary was used. We calculate the probabilistic contact matrices for each year (2003–2012) for each of the 1000 bootstrap samples and simulate an epidemic based on each sample. We then construct 95% confidence intervals around the model outcomes using the 2.5 and 97.5 percentiles of the estimates from the 1000 replications. Electronic supplementary material, figure S4 illustrates the ATUS sampling error propagating through the epidemic simulation.

## 3. Results

### (a) Additional time at home

Individuals increased their time spent at home in response to CDC confirmed cases by a statistically significant amount (table 1; table with results for all control variables provided in electronic supplementary material, table S3). We quantify sample uncertainty through 95% confidence intervals and model uncertainty by estimating several model specifications for robustness. We use the point estimate from model 3 that controls for state and month fixed effects, which suggests that people spent 2.38 additional minutes at home for every 1000 CDC confirmed cases with a 95% confidence interval of (0.278, 4.48). The avoidance response is statistically significant across model specifications (models 1–3 and the two additional models in the electronic supplemental material).

**Table 1.** Regression results for time spent at home.

	model 1	model 2	model 3
state fixed effects	x		x
month fixed effects		x	x
month×state fixed effects			x
coefficient estimates			
CDC reported cases	1.663 (0.944)*	2.378 (1.057)**	2.379 (1.072)**
Google media index	−22.33 (18.98)	−15.02 (19.61)	−17.66 (20.10)
extreme weather	30.88 (11.97)***	33.54 (11.95)***	34.47 (12.20)***

*p < 0.1; **p < 0.05; ***p < 0.01.

Our estimates suggest that the average person in the population spent an additional 22.11 min at home, with 95% confidence interval of (5.76, 33.57), at the peak of the epidemic when the CDC reported 9734 new cases nationally in a single week. This effect size is an average across the entire population, with some individuals likely to be spending substantially more time at home and others spending less. For comparison, the average individual spent 34.47 (10.56, 58.38) additional minutes at home during extreme weather events (e.g. snowstorms). Furthermore, we find no evidence that historically sensitive groups (persons 65+ years old and parents with children) engage in additional avoidance despite spending substantially more time at home, regardless of the epidemic state (electronic supplementary material, table S3, models 4 and 5).

**Figure 1.**
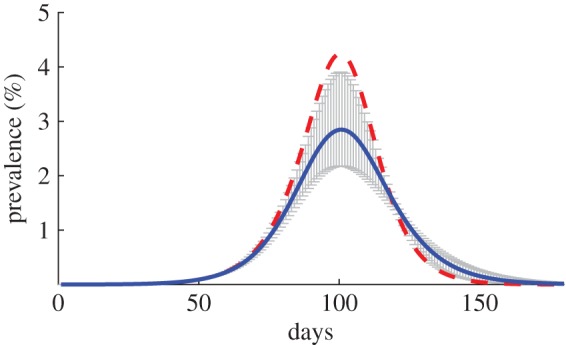
Simulated epidemic curves. The solid (blue) line indicates epidemic with avoidance behaviour and the dashed (red) line without, and the grey bars represent 95% confidence intervals. The simulations are based on the estimated 2.38 min reduction in time spent in public per thousand cases. The susceptible population is 4.1 × 10^6^, the recovery rate is 3 days and the infectivity parameter is chosen such that the basic reproduction number is 1.4.

We illustrate the potential epidemiological significance of this avoidance response by comparing two simulated epidemics: (i) when individuals fail to respond to the epidemic, and maintain a constant level of contacts (the standard assumption in most epidemic studies); and (ii) when individuals reduce their time spent in public by 2.38 (0.278, 4.48) min per thousand confirmed cases (model 3 in table 1). As prevalence of the infection rises, individuals that engage in avoidance gradually shift time in public into their household relative to the no-avoidance case. This is true despite the fact that the small fraction of infected households (in our model always less than 5% at a point in time) may not be safer than public. The substitution of relatively safe household time for time in public drives a wedge between the simulated epidemics with (dashed) and without (solid) avoidance behaviour (figure 1). At the peak of the simulated epidemic on day 92, individuals spend 22.11 min less in public with a confidence interval of (5.76, 33.57), which reduces the peak prevalence by 31% from 4.22% of the population without a behavioural avoidance model to 2.90% (2.18%, 3.90%) with the avoidance model. As the epidemic wanes, so too does the incentive to stay at home. The daily incidence during the last half of the infection is greater when individuals avoid infection early on because more of the population remains susceptible and avoidance behaviour fades. Nevertheless, by the end of the epidemic avoidance behaviour reduces the attack rate by a proportional 13% (2.99%, 21.03%) from 50% of the population without avoidance behaviour to 42.22% (37.46%, 48.29%) with avoidance, which is comparable with the simulated attack rate of A/H1N1 reported in [45].

### (b) Adjusting contact patterns

**Figure 2.**
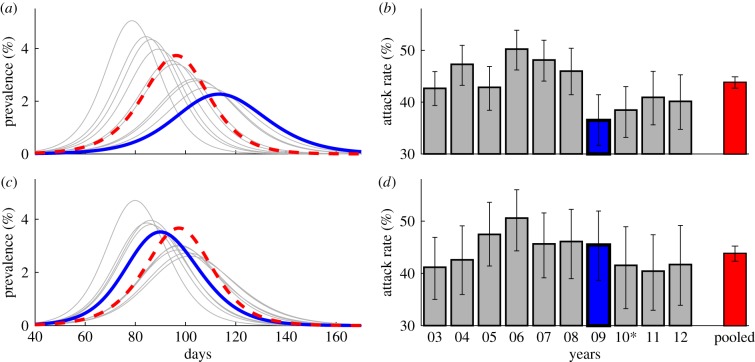
Simulated epidemic curves and cumulative cases based on contact matrices duringr (*a,b*) the epidemic period 20 April–20 Decembe and (*c,d*) the pre-epidemic period 1 January–19 April. (*a*,*c*) Percentage of the population infected by day. Solid (blue) lines are 2009, dashed (red) lines are non-2009 average and thin (grey) lines are non-2009 by year. (*b,d*) Cumulative number of infected and recovered individuals at the end of the epidemic where the bars indicate 95% confidence intervals. The asterisk indicates that the pandemic was not declared over until 23 June 2010 even though very few cases were reported in 2010.

Simulation results using the empirical PCMs suggest that individuals modified contacts during the A/H1N1 outbreak, reducing transmission rates and the impact of the epidemic relative to the average across all years other than 2009 (figure 2). During the outbreak period, peak prevalence falls from 3.76% (3.53%, 4.00%) of the population in the average simulation to 2.52% (1.81%, 3.32%) in 2009, a 33% decrease. This reduction in peak prevalence is comparable with the 31% decrease found by simulating the avoidance behaviour based on the regression results. The smaller epidemic in 2009 translates into a lower attack rate, 36.48% (31.62%, 41.45%), compared with the average case 43.81% (42.70%, 44.91%). Moreover, an epidemic based on contact patterns in the 2009 pre-outbreak period appears similar to the average across all years. The attack rate in 2009 is 45.45% (38.66%, 51.93%), whereas the attack rate in the average across all years is 43.80% (42.32%, 45.23%). These results and sensitivity analyses are contained in electronic supplementary material, table S4.

**Figure 3.**
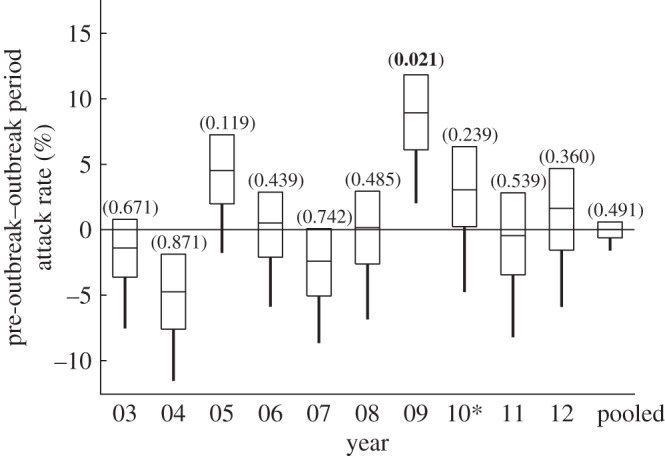
Comparing the epidemic and pre-epidemic simulations. The difference between simulated cumulative cases from the pre-epidemic period 1 January–19 April (figure 2*d*) and the epidemic period 20 April–20 December (figure 2*b*) with 95% lower confidence bound represented by bars and *p*-value of a one-sided hypothesis test with a null that the difference is less than or equal to zero. We reject the null hypothesis for 2009.

Figure 3 graphically presents a one-tailed *t*-test of the null hypothesis that the cumulative attack rate during the outbreak period does not exceed the cumulative attack rate during the pre-outbreak period and the associated *p*-values. 2009 is the only single year of the ten with a statistically significant result at *α* = 0.05, 5.88 percentage points with a *p*-value of 0.02. Tests for the other years are placebo tests. We would be surprised if the null were rejected for these tests. Moreover, multiple testing of placebos increases the probability of a false rejection (i.e. *p*-value of <0.05) to 0.37. Rejecting any one of these placebo tests would have weakened our result; however, the placebo test levels should be evaluated using a Bonferroni adjustment (adj. *α* = 0.006). Figure 3 also presents a pooled distribution, which can be compared with the 2009 result with a Kolmogorov–Smirnov test [46]. We reject the null hypothesis that the 2009 distribution is the same as the pooled distribution with a test statistic of *D* = 0.689, *p*-value of less than 0.001.

The simulation model based on the empirical PCMs disaggregates the population by age and household size. We find less behavioural heterogeneity across household size than across age groups. The household size heterogeneity that is present indicates that single-person households and large households of five or more suffer lower attack rates than households of two to four individuals. This finding is consistent with Cauchemez *et al*. [41] and may indicate that members of larger households spend more time at home. We provide more detail in the electronic supplementary material.

The regression model indicates that many factors influence how people spend their time. Because the empirical PCMs simply reflect probabilistic interactions between age groups, alone they do not reveal the mechanism responsible for the change in behaviour. However, the combined evidence from the simulations based on the empirical PCMs and the regression model, which does identify avoidance behaviour as an epidemiologically significant factor, suggests that people changed their behaviour during the A/H1N1 epidemic in a way that measurably affected epidemiological dynamics.

## 4. Discussion

We measure the extent to which Americans engaged in voluntary avoidance behaviour during the 2009 A/H1N1 epidemic and show that such behaviour is of epidemiologically meaningful magnitude. Our estimates derive from a national time-use survey conducted by the US Census Bureau nearly every day since 2003. We show that individuals spent on average 2.38 (0.278, 4.48) additional minutes at home for every 1000 CDC confirmed cases during the 2009 A/H1N1 epidemic. Moreover, simulations based on empirical contact matrices suggest that individuals adjusted behaviour in a manner that reduced contact time during the outbreak period in 2009, unlike the pre-outbreak period in 2009 or the outbreak period in any other year. These results are further supported by recent anecdotal evidence of avoidance behaviour in American churches frequented by immigrants from West Africa during the recent Ebola crisis [47].

Social distancing policies are an important public health tool for controlling epidemics, particularly during the early stages. However, the social and economic costs of social distancing policies imply that public health officials must weigh the costs and benefits of such measures to determine when to employ the social distancing policy [13,36]. Most research on social distancing policy attributes all behavioural response to the policy [48,49]. Our results provide empirical evidence that individuals respond to disease risk with behavioural shifts that are likely to be sufficiently large to influence the course of an epidemic. Therefore, emergency response plans—based on epidemic forecasts that neglect self-directed behavioural response—may prescribe costly measures to reduce transmission rates that would occur because of voluntary avoidance behaviour. It is also possible that poorly planned social distancing policies could counteract innate responses and ‘crowd out’ avoidance responses [7,50]. Furthermore, retrospective analysis of social distancing policies may appear beneficial when compared with a baseline forecast that did not account for feedbacks and adaptive avoidance behaviour. Including accurate self-directed adaptive behavioural responses in baseline models is imperative for those models to accurately guide public health policy.

We have described the epidemiological implications of uniform avoidance behaviour in a population. However, our control variables in the fixed-effects regressions make it possible to consider heterogeneity in avoidance behaviour across subpopulations (e.g. age classes). This heterogeneity may reflect variation in risk perception (the cost of avoidance) as well as the benefits of contact. The development of a heterogeneous mixing model with heterogeneous avoidance behaviour is beyond the scope of this work, but could have important implications for targeted public health policies [48–50].

Feedbacks between human behaviours and biological processes are challenging to measure, but are receiving great attention in public health, ecology, earth systems and sustainability. While many human–environmental feedbacks probably exist, the strength of these feedbacks is an empirical question. Our measure of the strength of feedback between adaptive human behaviour and epidemiological conditions suggests meaningful feedbacks that have implications for the study of infectious disease, the costs of infectious diseases and public health policy.

## Supplementary Material

Supplementary Materials

## Data accessibility

The American Time-Use Survey data files are available at http://www.bls.gov/tus/datafiles_0314.htm. Data from Google are available at https://www.google.com/trends/.
