# Supplementary material for: Measured voluntary avoidance behaviour during the 2009 A/H1N1 epidemic
Source: Proc Biol Sci. 2015 Nov 7;282(1818):20150814. doi: 10.1098/rspb.2015.0814 (PMC4650148; doi:10.1098/rspb.2015.0814)
Supplement: Supplementary Materials [file rspb20150814supp1.pdf]

Supplementary Materials:

Additional Data Description

Supplementary Table S1 contains the number of survey respondents and the mean daily minutes spent at home by year. The mean daily time spent at home is relatively stable throughout the sample ranging from 461 minutes in 2005 to 481 minutes in 2012. In the inaugural year of the ATUS, the BLS surveyed over 20,000 individuals. According to the ATUS documentation[1], the BLS reduced the sample size after 2003 to comply with the survey budget.

Extreme weather is used to control for additional time spent at home to avoid weather rather than flu risk. Extreme weather data is tracked by the NOAA's Storm Data available through the National Climatic Data Center (NCDC) at the county level throughout the US. The Storm Data documents cases of weather activity that falls into one of the following three categories[2]:

a. The occurrence of storms and other significant weather phenomena having sufficient intensity to cause loss of life, injuries, significant property damage, and/or disruption to commerce;

b. Rare, unusual, weather phenomena that generate media attention, such as snow flurries in South Florida or the San Diego coastal area; and

c. Other significant meteorological events, such as record maximum or minimum temperatures or precipitation that occur in connection with another event.

We construct a dummy variable associated with each ATUS respondent equal to one if any of these events was recorded in a particular county on a particular day. The information included in

---

[1] http://www.bls.gov/tus/atususersguide.pdf

[2] http://www.nws.noaa.gov/directives/sym/pd01016005curr.pdf (p4)

the Storm Data set includes a short description of the event, start date of the weather, end date of the weather in addition to state, and county and city identifiers of where the weather was taking place. We match the location of the event to the respondent's home location with precision to the city or county level depending on level of geographic censorship in the ATUS.

We collect Google search data to represent a subjective measure of risk. The measure is subjective because it is based on news coverage and general interest in the population rather than true risk as measured by the disease prevalence. Google monitors users' search volume of specific key words and the occurrence of key words in media headlines from a variety of sources. Google presents the information in the form of a normalised index from 0-100 where 100 represents the most search volume over a given time interval. We collect Google Trends for the search terms "swine flu" or "h1n1" in the U.S. only between the dates April 19, 2009 to April 04, 2010. Google search volume peaks (100) during the week April 19-26, 2009 (Supplementary Figure S1). We use Google Trends to measure media attention rather than Google Flu Trends, a proprietary prediction tool offered by Google, because our objective is to measure the population's discussion about infection risk, which is not necessarily correlated with actual prevalence.

Regression Model

In addition to regression models 1-3 described in the main text, we include two additional models in the supplementary material. Model 4 includes a dummy for age 65 and older individuals and an interaction of that dummy variable with the number of laboratory-confirmed cases to test whether avoidance behaviour was stronger among more sensitive individuals. Model 5 includes a similar set of terms for parents spending time with children. The ATUS does not survey individuals less than 15 years; however, respondents specify which family members were

present during each activity and their demographic information. We use parents' time at home with children as a proxy for the behaviour of children less than 15 years old. Model 6 includes a third set of interactions between parents with children and Saturday to further investigate avoidance behavior on the weekend.

Regression Results

We present the full set of parameter estimates for all variables used in the regression model in Supplementary Table S3. The constant coefficient represents the time spent at home by the baseline individual represented by the omitted categories or binary outcomes in the regression (e.g., less than college degree, other race, female, not extreme weather). The baseline respondent spends 335.1 (model 1) to 357.9 (model 2) minutes at home on Monday in January in Alabama (excluding sleep and other personal activities). The coefficients on laboratory confirmed cases ($CASES$) is statistically different from zero in models 1-6, which suggests that the result is robust to various model specifications.

In all models, individuals with a spouse or partner spend more time at home. People spend more time at home as their age increases, but at a decreasing rate. Similarly, people tend to spend more time at home during the weekend (40-60 minutes). We find that ill and disabled individuals do not spend any statistically significant additional time at home. We do find that students, employed individuals, and those with a college degree and beyond spend much less time at home. The estimated coefficient on the indicator for extreme weather conditions indicates that individuals spent between 30 and 34 additional minutes at home during an adverse weather event. We would expect this effect to provide an approximate upper bound on avoidance behaviour to H1N1 because it occurs in situations where individuals have a very strong incentive

to stay home. That is, a severe storm effectively forces people to stay indoors whereas they are not forced to stay indoors by health risk.

The $MEDIA$ coefficient indicates that individuals spent less time at home during the peak of the Google flu-related searches. This is not entirely surprising given the divergence between public attention to the epidemic and media attention suggested by figure 2. However, this effect is not statistically different from zero at the 10% level. In contrast, the statistically significant coefficients on $CASES$ suggest that people respond to the objective measure of risk.

We expect seniors to be more responsive to flu risk because they have a higher probability of becoming seriously ill or dying, and because they are less likely to be time constrained by work and caring for young children. The $ELDER$ variable in model 4 indicates that individuals 65 years and older spend 32 additional minutes at home with a 95% confidence interval of [12.20, 51.08] regardless of infection risk. The interaction of $ELDER$ with $CASES$ suggest that seniors spent an additional 14 [-27.65, 56.23] minutes at home in response to increased flu risk during the week with the most confirmed H1N1 cases. However, the interaction is not statistically significant, due in part to the smaller number of seniors in the ATUS sample, and perhaps because these individuals already spend more time at home creating a small margin on which to adjust behavior.

Model 5 adds an interaction between $CASES$ and whether the respondent spent time with their own children at some point during the day. Similar to seniors, young children are more susceptible to negative health consequences of contracting the flu. Therefore, we would expect their parents to have a greater incentive to take actions to reduce their probability of contracting the flu. This intuition is not mirrored by our results. The estimated coefficient is negative. This may indicate that parents with young children have less flexibility to adjust their schedules, that

they did not perceive the health risks to be heightened, or that having children leads people to behaviour fatalistically [1] with respect to flu.

Model 6 interacts parent's activities with children and $CASES$ along with a dummy variable Saturday. People spend an average of 54.57 minutes at home on Saturday. Parents with children spend an average of 16.58 additional minutes at home on Saturday whereas individuals without children spend 12.64 minutes less at home. Importantly, interacting parent's avoidance response with Saturday causes the negative coefficient to be statistically insignificant while the population average avoidance response is 3.345 per 1000 cases and is statistically significant. Therefore, we find no conclusive evidence of additional avoidance behavior by parents with children.

Our model accounts for approximately 20% of the variation in time spent at home ($R^2 = 0.20$ in models 3-6).The magnitude and statistical significance of estimates on employment, weather, and day of week demonstrate that our covariates capture certain critical factors that influence time-use patterns.

Epidemic Simulations Adjustment

Laboratory confirmed cases represent only a fraction of total cases suspected in the population [2]. Reed et al. [2] estimated that 43,677 laboratory confirmed cases through July 23, 2009 represented between 1.8 and 5.7 million cases in the population. Using the most conservative estimate, we assume that 2.4% of simulated cases are confirmed by laboratory testing. Furthermore, confirmed cases are measured at the national level so we scale the simulated number of infected individuals (based on Phoenix MSA) to the national level by multiplying daily prevalence by 3.47 (9,734 national cases/2,800 Phoenix MSA cases during the peak of the epidemic in the third week of October [3]). The product of the proportion of laboratory-

confirmed cases and the proportion of national cases to those reported in Phoenix MSA yields $\phi = 8.33\%$.

Sensitivity of Household Contact Scalar ($\boldsymbol{\alpha}$)

We investigate the sensitivity of our results to the assumption of $\alpha = 1$ made on the basis of empirical studies of household transmission during the 2009 A/H1N1 outbreak. We re-simulate the model assuming α =3 and α =5. We keep the infectivity parameter constant in each simulation δ =1.4 10^3, which prevents the model from generating the number of cases observed in the data. Nevertheless, the simulations provide intuition about the impact of increasingly infectious household contacts. The peak prevalence and attack rates from the avoidance model and the empirical PCM model are reported in Supplementary Table S4.

As α increases, time spent engaging in household contacts is more infectious in households with at least one infected person. If α=3, household contacts are three times more infectious per minute than public contacts. The attack rate rises in the simulations with and without avoidance to over 80% of the population, but the percent change between the two simulations with and without avoidance falls by 7.9 percentage points. If α=5, the attack rates with and without avoidance reach nearly 100% of the population. Under such a severe epidemic, most households become infectious, which exacerbates the effect of increased infectivity of household contacts.

Probabilistic Contact Matrix (PCM).

We compare simulated epidemic outcomes in each year 2003-2012 using an age-household size model based on PCMs derived from ATUS data. Following Zagheni et al. [4], let $\ell \in L$ denote a public location reported in the ATUS. Public locations include: School; Restaurant or bar; Place of worship; Grocery store; Mall and other stores; Gym and Health club; Personal Services e.g.,

laundry, beauty salon; Public building, library and post office; Public bus; Subway and train; Boat and ferry; Airplane; Office building and bank, Factory/Manufacturing center, Hospital and health care center, Workplace not elsewhere classified; Public location not elsewhere classified. Let $m \in [0{,}1440]$ denote an index of time (minutes in a 24 hour period). Let $k \in P$ denote an age-household size group (P ={0-4, 5-12, 13-17, 18-24, 25-49, 50-64, 65+}×{1, 2, 3, 4, 5+}) within which all members mix homogeneously. These groupings strongly reflect United States schooling and labour behaviours. In the United States children start school at approximately age five. In many states children become eligible for part-time work and start secondary school around 14 years-old . Most children finish secondary education at age 18. Between ages 18 and 24 Americans are often entering the labor force or attending college or university. This is transitional age for many Americans. Working age adults are broken into two groups. Sixty-five is used as a retirement age. Then, $g_k^{\ell m}$ is the number of individuals in age group k at location ℓ at minute m. The exposure of group k to group $j \neq k$ is proportional to the size of each population at location ℓ at minute m and is given by

$$z_{kj}^{\ell m} = g_k^{\ell m} \times \frac{g_j^{\ell m}}{\sum_l^K g_l^{\ell m}}$$

The total time of public exposure on an average day between groups k and j at location ℓ is $z_{kj}^{\ell} = \sum_{m=0}^{1440} z_{kj}^{\ell m}$ and the total time of exposure between groups k and j at all public locations is $z_{kj} = \sum_{\ell \in L} \sum_{m=0}^{1440} z_{kj}^{\ell m}$. The exposure matrix, $\boldsymbol{Z}$, has dimensions $(K, K, L, 1440)$ and is symmetric in (K, K)-space, by construction. The structure of the exposure matrix implies a symmetric contact matrices such that $z_{kj} = p_{kj} w_j = p_{jk} w_k$ where p_{kj} is the contact rate between population k and individual j, and w_j is the population of group j (i.e., a contact between groups k and j is necessarily a contact between groups j and k) [5]. However, the relevant measure is

the contact between an individual in group j and the whole of population of group k. Therefore, we divide the columns of the matrix $\boldsymbol{Z}$ by population w_j, given by the sample weights in the ATUS, to form the PCM. A column in the PCM sums to the total number of minutes an average individual in group j spends in public. All contact matrices and the code written to generate them in Matlab are available upon request from the authors.

The ATUS surveys a subset of respondents to the Current Population Survey (CPS), which interviews only individuals 15 years and older. However, ATUS asks respondents to report information on all family members and whether any family members were present during an activity. We use the activity and location information on children with their parents (primary ATUS respondents) in public to construct a partial measurement of children's public contact patterns. Children younger than 13 years old are rarely in public microenvironments without an adult (see Supplementary Figure S2). School contacts are largely omitted from this calculation because parents generally leave their children at school for long periods during the weekday.

The National Health and Activity Patterns Survey (NHAPS) was conducted from 1992-1994 and collected time use information on children by microenvironment (e.g., school, home) at minute resolution. We use the NHAPS to construct children's contact patterns at school. We then derive an index for children's time at school based on 15-17 year olds from the ATUS and use that index to rescale school contact patterns from the NHAPS. The base of the index is the average time spent at school from 2003-2012 omitting 2009. The index is constructed separately for the pre-outbreak and outbreak periods. Indices are created for each bootstrap sample and used to adjust the contacts at school in each bootstrap simulation.

Supplementary Figures S3 depict the household and public PCMs during the outbreak period averaged across 2003-2012 as heat maps with associated population distributions directly

above. Panel a) illustrates the contact patterns by age and household size in the household environment. Segmenting the population by household size allows our population-based model to capture intra-household transmission but importantly, prevents inter-household transmission in the home environment. The household PCM clearly shows interaction between children and their parents or guardians in households of three or more, and strong affinity mixing between adults in two-person households. The contact with children in single-person households is a result of children visiting parents in cases where the children do not live with the parent surveyed regularly. The population density just above the heat map shows that this population is small, so that this contact time has almost no impact on the simulation results.

Panel b) illustrates that contact time in public are heterogeneous across age groups. Adults primarily contact other adults in public, especially those in smaller households. Children, who are generally in households with three or more people, experience most of their contact time with other children at school. Together, the household and public PCMs capture a rich set of epidemiologically meaningful contact patterns.

Sampling Uncertainty

Like any sampling method, the ATUS samples include uncertainty, which we propagate through our simulations. In the main text, we illustrate sample uncertainty with 95% confidence bars around cumulative attack rate in Figure 2. In Supplementary Figure S4, we illustrate the sampling uncertainty associated with the 2009 ATUS survey data during the outbreak period (panel A) and pre-outbreak period (panel B) on each day of the epidemic (daily frequency). During the outbreak period (panel A), the peak prevalence of the epidemic curve based on the average PCM lies outside of the 95% confidence interval of the 2009 epidemic curve. This figure suggests that our result is not due to sampling uncertainty in the ATUS. In contrast, panel

B illustrates that the 2009 and average epidemic curves are statistically indistinguishable during the pre-outbreak period.

Supplementary Figure S4 shows that the sampling errors are asymmetric near the peak prevalence. The pattern of uncertainty is consistent across both simulations because of the mechanics of the model and the source of the uncertainty. We hold the contact matrix constant during each simulation. When a particular Monte Carlo sample from the ATUS yields a contact matrix with high contact minutes (i.e., respondents that happen to collocate more frequently), the simulated epidemic will spread through the population faster and the peak prevalence will occur earlier. The Matlab code and bootstrap simulation data are available upon request.

References

1. Kremer, M. 1996 Integrating Behavioral Choice into Epidemiological Models of AIDS. *The Quarterly Journal of Economics* **111**, 549–573. (doi:10.2307/2946687)

2. Reed, C., Angulo, F. J., Swerdlow, D. L., Lipsitch, M., Meltzer, M. I., Jernigan, D. & Finelli, L. 2009 Estimates of the Prevalence of Pandemic (H1N1) 2009, United States, April–July 2009. *Emerging Infectious Diseases* **15**, 2004–2007. (doi:10.3201/eid1512.091413)

3. Arizona Department of Health Services In press. Arizona - Weekly Influenza Summary MMWR Week 31-32 (August 1, 2010 - August 14, 2010).

4. Zagheni, E., Billari, F. C., Manfredi, P., Melegaro, A., Mossong, J. & Edmunds, W. J. 2008 Using Time-Use Data to Parameterize Models for the Spread of Close-Contact Infectious Diseases. *Am. J. Epidemiol.* **168**, 1082–1090. (doi:10.1093/aje/kwn220)

5. Wallinga, J., Teunis, P. & Kretzschmar, M. 2006 Using Data on Social Contacts to Estimate Age-specific Transmission Parameters for Respiratory-spread Infectious Agents. *Am. J. Epidemiol.* **164**, 936–944. (doi:10.1093/aje/kwj317)

6. Zar, J. H. 1996 *Biostatistical Analysis*. 3rd edition. Upper Saddle River, N.J: Prentice Hall College Div.

7. Holm, S. 1979 A Simple Sequentially Rejective Multiple Test Procedure. *Scandinavian Journal of Statistics* **6**, 65–70.

Supplementary Table S1. Descriptive statistics of time spent at home by ATUS respondents 2003-2012.

	Surveys	Mean Time at Home (Minutes)	St. Dev.
2003	20,720	462.37	266.88
2004	13,972	468.34	267.65
2005	13,038	461.88	266.36
2006	12,941	463.49	268.17
2007	12,248	463.67	269.87
2008	12,723	472.05	270.55
2009	13,133	480.29	268.51
2010	13,259	475.95	270.91
2011	12,478	476.20	271.50
2012	12,443	481.71	272.50
2003-2012	136,955	470.10	269.21

Supplementary Table S2. Summary Statistics

	Mean	St. Dev.	Min	Max
Time at Home (Minutes)	470.10	269.21	0	1400
CASES (1000)	0.729	1.660	0	9.735
Age (Years)	45.867	17.512	0	85
MEDIA (index 0-1)	0.027	0.087	0	1

Frequency Tables

Regression Variables		PCM Variables	
Male	43.1%	Age:	
Race:		0-4	7%
White	81.4%	5-12	11%
Black	13.3%	13-17	7%
Other Race	5.3%	18-24	9%
Hispanic	13.1%	25-49	35%
Partner Present	52.8%	50-64	18%
Education:		65+	13%
Less than College	38.4%	Household Size:	
College Degree	48.9%	One	11%
Advanced Degree	12.7%	Two	26%
Current Student	11.0%	Three	18%
Currently Employed	63.1%	Four	23%
Manual Labor	7.8%	Five or more	20%
Ill/Disabled	0.3%		
Extreme Weather	1.3%		
Survey Day:			
Holiday	1.6%		
Monday	9.3%		
Tuesday	9.2%		
Wednesday	9.4%		
Thursday	9.6%		
Friday	9.9%		
Saturday	26.0%		
Sunday	26.6%		

Supplementary Table S3. Fixed effect regression results for time spent at home.

	(1)	(2)	(3)	(4)	(5)	(6)
State fixed effects	x		x	x	x	x
Month fixed effects		x	x	x	x	x
Month*State fixed effects			x	x	x	x
	Coefficient Estimates of fixed effects models					
	(1)	(2)	(3)	(4)	(5)	(6)
CASES (1000)	1.663*	2.378**	2.379**	2.114*	3.357***	3.345***
	(0.944)	(1.057)	(1.072)	(1.129)	(1.219)	(1.218)
Elderly (65+ yrs)				31.64***		
				(7.924)		
CASES *Elderly				1.468		
				(2.198)		
Child (<18 yrs)					15.50***	16.58***
					(3.490)	(3.927)
Child*Saturday						-12.64**
						(6.369)
No Child*Saturday						16.58***
						(3.927)
CASES*Child					-3.263*	-2.084
					(1.690)	(1.867)
CASES*Child*Saturday						-3.809
						(3.084)
MEDIA	-22.33	-15.02	-17.66	-17.17	-17.06	-16.80
	(18.98)	(19.61)	(20.10)	(20.07)	(20.12)	(20.11)
Extreme Weather	30.88***	33.54***	34.47***	34.36***	34.46***	34.52***
	(11.97)	(11.95)	(12.20)	(12.20)	(12.21)	(12.21)
Holiday	5.931	5.240	6.910	7.029	5.237	5.757
	(11.93)	(11.96)	(11.93)	(11.93)	(11.96)	(11.95)
Tuesday	-6.286	-6.418	-5.172	-5.202	-5.240	-5.194
	(5.805)	(5.792)	(5.875)	(5.871)	(5.867)	(5.867)
Wednesday	-11.51**	-11.67**	-10.72*	-10.71*	-10.76*	-10.66*
	(5.738)	(5.730)	(5.818)	(5.817)	(5.809)	(5.809)
Thursday	-19.65***	-19.25***	-18.20***	-18.38***	-18.46***	-18.47***
	(5.696)	(5.680)	(5.786)	(5.784)	(5.779)	(5.778)
Friday	-16.08***	-16.08***	-14.60**	-14.66**	-15.16***	-15.20***
	(5.728)	(5.708)	(5.797)	(5.796)	(5.795)	(5.796)
Saturday	41.23***	41.49***	41.27***	41.25***	39.76***	54.57***
	(4.972)	(4.957)	(5.024)	(5.022)	(5.042)	(6.711)
Sunday	59.69***	60.07***	59.70***	59.66***	57.87***	57.59***
	(4.835)	(4.818)	(4.892)	(4.891)	(4.919)	(4.927)

Supplementary Table S3 cont. Fixed effect regression results for time spent at home.

	(1)	(2)	(3)	(4)	(5)	(6)
State fixed effects	x		x	x	x	x
Month fixed effects		x	x	x	x	x
Month*State fixed effects			x	x	x	x
	Coefficient Estimates of fixed effects models					
College Degree	-5.780*	-5.493*	-5.707*	-6.141*	-5.773*	-5.797*
	(3.123)	(3.101)	(3.152)	(3.154)	(3.151)	(3.151)
Advanced Degree	-20.75***	-19.84***	-21.74***	-22.22***	-21.96***	-21.97***
	(4.563)	(4.527)	(4.612)	(4.612)	(4.612)	(4.612)
Hispanic	-13.37***	-14.79***	-13.83***	-14.12***	-14.66***	-14.71***
	(4.222)	(3.899)	(4.266)	(4.266)	(4.271)	(4.271)
White	-6.797	-4.978	-7.507	-7.540	-7.311	-7.349
	(6.251)	(6.080)	(6.278)	(6.277)	(6.276)	(6.276)
Black	-24.03***	-23.43***	-25.36***	-25.29***	-24.86***	-24.93***
	(7.280)	(7.049)	(7.308)	(7.306)	(7.305)	(7.306)
Ill/Disabled	-8.718	-6.528	-12.42	-10.82	-13.10	-13.17
	(23.10)	(23.02)	(22.81)	(22.89)	(22.89)	(22.88)
Age	4.229***	4.191***	4.234***	5.637***	4.109***	4.105***
	(0.484)	(0.483)	(0.488)	(0.582)	(0.490)	(0.490)
Age2	-0.019***	-0.018***	-0.019***	-0.038***	-0.016***	-0.016***
	(0.005)	(0.005)	(0.005)	(0.007)	(0.005)	(0.005)
Male	-18.70***	-18.73***	-18.86***	-18.76***	-17.26***	-17.18***
	(2.787)	(2.783)	(2.809)	(2.808)	(2.833)	(2.834)
Employed	-144.5***	-144.6***	-144.5***	-143.8***	-143.7***	-143.7***
	(3.367)	(3.358)	(3.391)	(3.396)	(3.396)	(3.395)
Manual Labor	-0.496	-1.209	-1.331	-1.311	-1.326	-1.407
	(5.306)	(5.290)	(5.394)	(5.395)	(5.393)	(5.391)
Student	-34.36***	-34.70***	-35.69***	-33.42***	-33.79***	-33.85***
	(5.267)	(5.282)	(5.368)	(5.390)	(5.375)	(5.376)
Partner	42.99***	43.04***	43.20***	42.27***	40.33***	40.38***
	(2.942)	(2.935)	(2.961)	(2.968)	(3.049)	(3.050)
Constant	335.1***	355.6***	357.9***	333.7***	355.0***	354.9***
	(17.15)	(13.47)	(47.07)	(47.45)	(47.17)	(47.26)
R2	0.176	0.176	0.195	0.196	0.196	0.196
Observations	27,091	27,091	27,091	27,091	27,091	27,091

Supplementary Table S4. Sensitivity of results from homogeneous mixing model and heterogeneous age-household size model. All peak prevalence and attack rate results are in percent.

Result	$\alpha=1$	95% CI	$\alpha=3$	95% CI	$\alpha=5$	95% CI
Homogeneous mixing model						
Peak Prev. w/ Av.	2.9	(2.18, 3.91)	14.93	(7.57, 19.82)	55.25	(46.51, 62.09)
Peak Prev. w/o Av.	4.22	-	20.34	-	44.16	-
% Change Peak Prev	31.2	(7.46, 48.27)	26.58	(2.56, 62.76)	-25.1	(-40.59, -5.30)
Atk. Rt. w/ Av.	42.22	(37.46, 48.29)	82.66	(65.49, 89.45)	99.98	(99.81, 100.00)
Atk. Rt. w/o Av.	50	-	89.5	-	99.59	-
% Change Atk. Rt.	15.55	(3.42, 25.07)	7.65	(0.06, 26.83)	-0.39	(-0.41, -0.22)
Age-household size model						
Pre-Outbreak Period						
Peak Prev. 2009	4.16	(2.82, 5.70)	17.94	(12.46, 22.49)	38.3	(35.26, 40.73)
Peak Prev. Avg	3.71	(3.41, 4.01)	16.61	(15.24, 17.92)	38.18	(37.06, 39.20)
Attack Rate 2009	45.45	(38.66, 51.93)	83.14	(75.02, 87.82)	94.58	(94.02, 95.13)
Attack Rate Avg	43.8	(42.32, 45.23)	82.27	(80.41, 83.93)	94.94	(94.76, 95.13)
Outbreak Period						
Peak Prev. 2009	2.52	(1.81, 3.32)	11.33	(6.83, 15.92)	35.72	(33.12, 38.03)
Peak Prev. Avg	3.76	(3.53, 4.00)	16.76	(15.87, 17.70)	38.37	(37.36, 39.21)
Attack Rate 2009	36.48	(31.62, 41.45)	71.86	(59.67, 81.07)	94.02	(93.43, 94.52)
Attack Rate Avg	43.81	(42.70, 44.91)	82.46	(81.21, 83.65)	95	(94.88, 95.13)
Difference 2009	8.97	(2.03, ~)	11.29	(1.65, ~)	0.57	(-0.06, ~)

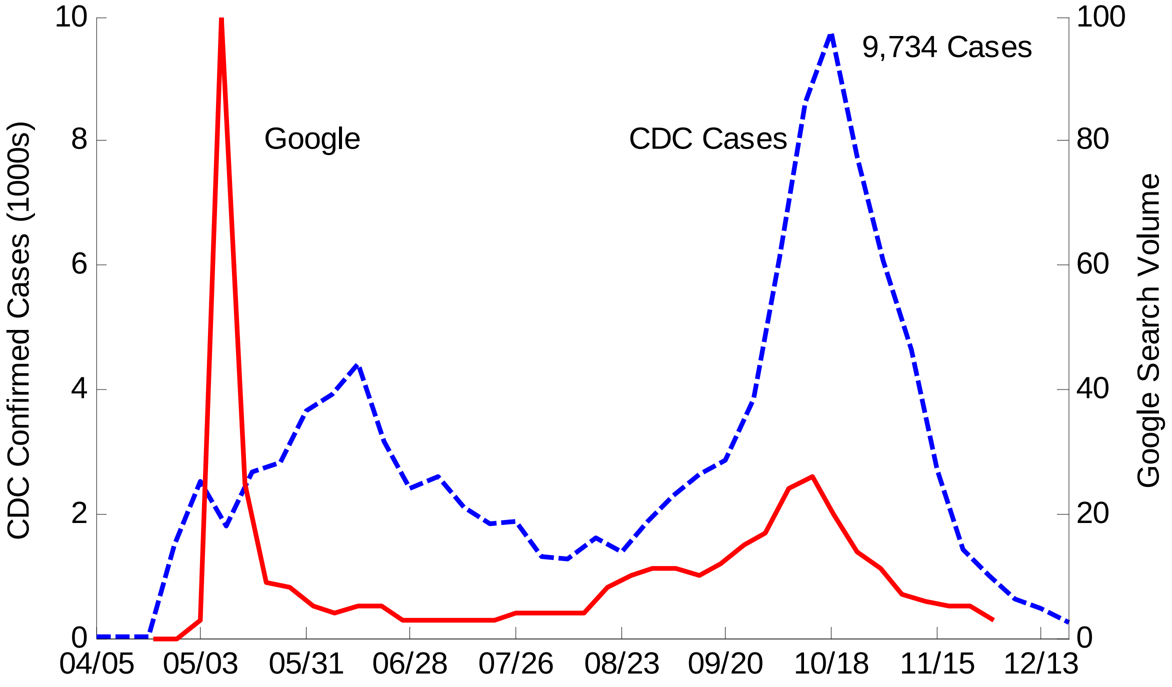

Supplementary Figure S1. Weekly number of CDC reported cases of H1N1 (dashed blue) and Google search volume index (solid red) over the outbreak period April 20, 2009 – December 20, 2009. The horizontal axis displays dates in one week increments with the starting date in the format "mm/dd".

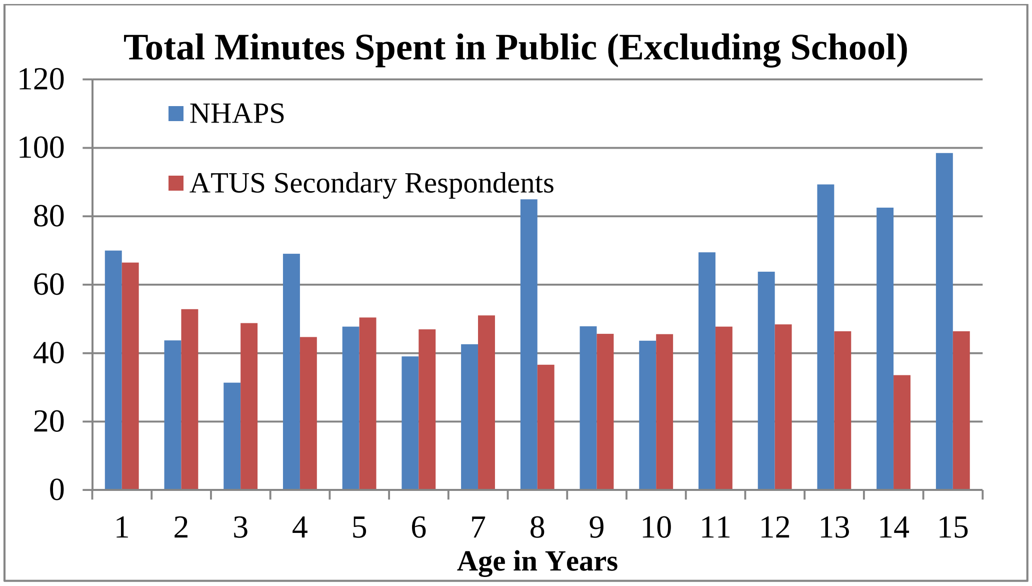

Supplementary Figure S2. Comparison of time spent in public using the NHAPS data from 1992-1994 and children reported with primary ATUS respondents (age 15 and older).

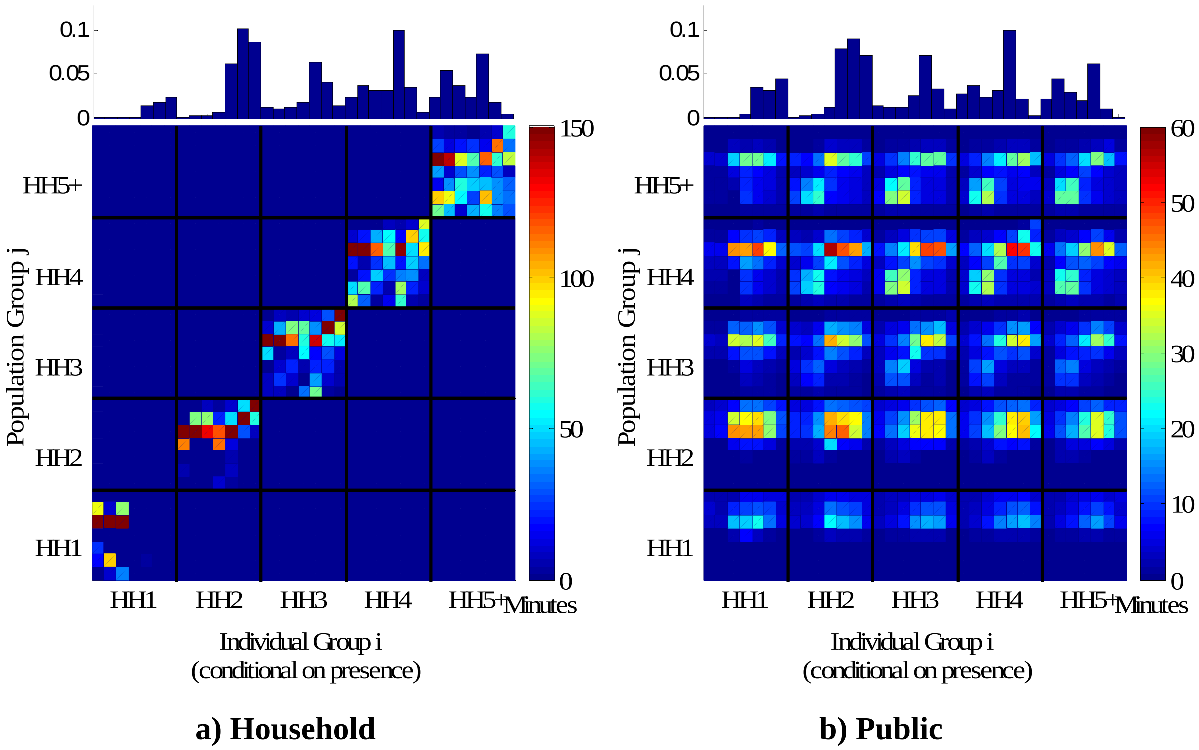

Supplementary Figure S3. Household and public PCMs with empirical population distributions above. Dark lines denote the five household size groups. Each household size category consists of the seven age groups for a total of 35 groups (P ={0-4, 5-12, 13-17, 18-24, 25-49, 50-64, 65+}×{1,2,3,4,5+}), note these bins are not of equal width. A cell represents the number of contact minutes an individual in group i interacts with the population of group j. The vertical sum of cells in a single column is equal to an individual's total contact minutes. The vertical axis of the population distribution (bar chart) is group i percent of the population. Note, these are the transpose of $\boldsymbol{C}$ described in the main text.

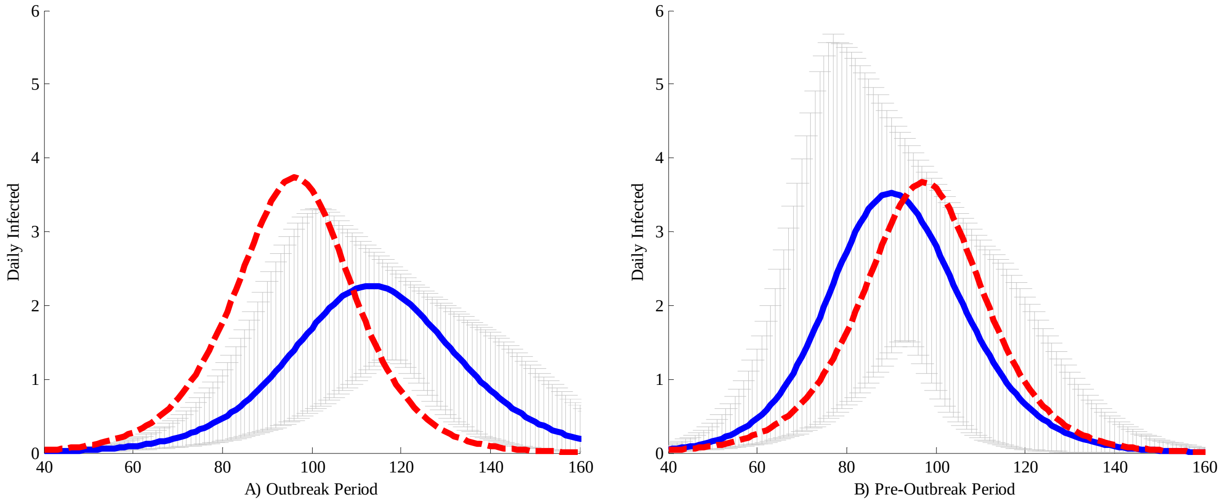

Supplementary Figure S4. Simulated epidemic curves based on contact matrices for 2009 (solid blue) and the average of all non-2009 years (dashed red) during the outbreak period (panel A) and the pre-outbreak period (panel B). The bars represent the 95% confidence interval of the 2009 curve.
